# Toxic-Metal-Induced Alteration in miRNA Expression Profile as a Proposed Mechanism for Disease Development

**DOI:** 10.3390/cells9040901

**Published:** 2020-04-07

**Authors:** David R. Wallace, Yasmeen M. Taalab, Sarah Heinze, Blanka Tariba Lovaković, Alica Pizent, Elisavet Renieri, Aristidis Tsatsakis, Ammad Ahmad Farooqi, Dragana Javorac, Milena Andjelkovic, Zorica Bulat, Biljana Antonijević, Aleksandra Buha Djordjevic

**Affiliations:** 1 School of Biomedical Science, Oklahoma State University Center for Health Sciences, Tulsa, OK 74107, USA; david.wallace@okstate.edu; 2 Forensic Medicine and Clinical Toxicology Department, Faculty of Medicine, Mansoura University, Dakahlia Governate 35516, Egypt or; 3 Institute of Forensic and Traffic Medicine, University of Heidelberg, Voßstraße 2, 69115 Heidelberg, Germany; Sarah.Heinze@med.uni-heidelberg.de; 4 Analytical Toxicology and Mineral Metabolism Unit, Institute for Medical Research and Occupational Health, Ksaverska cesta 2, 10 000 Zagreb, Croatia; btariba@imi.hr (B.T.L.); apizent@imi.hr (A.P.); 5 Centre of Toxicology Science and Research, University of Crete, School of Medicine, 71601 Heraklion, Greece; e.renieri@med.uoc.gr (E.R.); tsatsaka@uoc.gr (A.T.); 6 Institute of Biomedical and Genetic engineering, 54000 Islamabad, Pakistan; ammadfarooqi@rlmclahore.com; 7 Department of Toxicology “Akademik Danilo Soldatović”, University of Belgrade-Faculty of Pharmacy, Vojvode Stepe 450, 11221 Belgrade, Serbia; dragana.javorac@pharmacy.bg.ac.rs (D.J.); millena.andjelkovic@gmail.com (M.A.); zorica.bulat@pharmacy.bg.ac.rs (Z.B.); biljana.antonijevic@pharmacy.bg.ac.rs (B.A.)

**Keywords:** miRNA, gene expression, cadmium, lead, arsenic, mercury, manganese, cancer, neurodegenerative diseases (NDDs), epigenetic modification

## Abstract

Toxic metals are extensively found in the environment, households, and workplaces and contaminate food and drinking water. The crosstalk between environmental exposure to toxic metals and human diseases has been frequently described. The toxic mechanism of action was classically viewed as the ability to dysregulate the redox status, production of inflammatory mediators and alteration of mitochondrial function. Recently, growing evidence showed that heavy metals might exert their toxicity through microRNAs (miRNA)—short, single-stranded, noncoding molecules that function as positive/negative regulators of gene expression. Aberrant alteration of the endogenous miRNA has been directly implicated in various pathophysiological conditions and signaling pathways, consequently leading to different types of cancer and human diseases. Additionally, the gene-regulatory capacity of miRNAs is particularly valuable in the brain—a complex organ with neurons demonstrating a significant ability to adapt following environmental stimuli. Accordingly, dysregulated miRNAs identified in patients suffering from neurological diseases might serve as biomarkers for the earlier diagnosis and monitoring of disease progression. This review will greatly emphasize the effect of the toxic metals on human miRNA activities and how this contributes to progression of diseases such as cancer and neurodegenerative disorders (NDDs).

## 1. Introduction

### 1.1. Toxic Metals

The toxic effect of heavy metals such as cadmium (Cd), arsenic (As), lead (Pb), mercury (Hg), and manganese (Mn) is a matter of global concern. Although these metals are naturally occurring elements found in the earth’s crust, their concentrations in soil, air, and water have increased to exceed natural occurrence levels as a result of metal mining and manufacturing, agriculture, and other anthropogenic activities. These metals are utilized during a variety of manufacturing processes and often comprise the majority of the environmental pollutants [1]. Once released into the environment, these pollutants cannot be destroyed but rather are altered chemically, changing their bioavailability and toxicity. Ultimately, metals can contaminate the food chain, groundwater, drinking water, air, and soil [2]. Metals that end up in the groundwater and soil can undergo active uptake into plants, entering the food system via crops [3]. Toxic metals are further found as a contaminant in fertilizer, which in turn can enter the food cycle [4,5,6]. The effect of toxic metal contamination on crop production has been well documented [6,7,8,9,10,11]. Various studies have shown that the general population is exposed to these chemicals through different sources of exposure [12,13,14,15,16,17]. Hence, the data on their exact mechanisms of toxicity are of utmost importance for performing knowledge-based risk assessment. Here, any pertinent data from human clinical and epidemiological studies are used to determine if there are associations between the agent and disease occurrence [18].

The effect of the environmental metal toxicants on the epigenome has attracted a considerable interest in the past few decades. Epigenetics refers to the modification of gene expression through changes in the chemical makeup of nucleotides or the associated histone proteins rather than alteration in the genetic code or DNA sequence itself. Three common alterations have been studied, including DNA methylation, histone modifications, and noncoding RNA expression. However, epigenetic modifications are not generally limited to these types of alterations. Incorporating the toxic-metal-induced epigenetic alterations as informative factors in the risk assessment process is now rather fundamental given the role of epigenetic alterations in regulating gene and accordingly protein expression [19].

### 1.2. Toxicoepigenomics and Environmentally-Associated Diseases

With the great evolution of genetic/molecular biology, several high-throughput profiling technologies such as genomics, transcriptomics, proteomics, and metabolomics have been developed, among which toxicogenomics combines toxicology with these approaches to analyze the gene expression profile of several thousand genes aiming to identify changes associated with drug-induced toxicities [20]. In the last two decades, epigenetic alterations have shown to play a role in the transcriptional processes that regulate gene expression. Therefore, the field of toxicoepigenomics, which studies the relationship between epigenetic modifications and disease status in response to exposure to environmental contaminants and toxic agents, is now at the forefront of the environmental health science [19].

In this domain, if environmental exposure to various toxic agents progresses to a disease state through epigenetic alterations, the adverse health events will be predicted based upon gene expression profile. Concomitantly, factors regulating gene expression may also be used to predict these same outcomes. Therefore, incorporating epigenetic data into the risk assessment process would be of a great value given to the role of epigenetic regulation in gene expression upon exposure to toxicants. Epigenetic alterations can be used as biomarkers of the effect in response to exposure to environmental toxicants. These genetic biomarkers can be used additionally as predictors of the disease when such epigenetic marks are associated with differential gene expression. These alterations may be stable in the context of detection when they are heritable, predicting possible inherited gene expression changes in response to maternal toxicant exposure [21].

Furthermore, the involvement of the epigenetic data in disease progression necessitates determination of four main parameters: Are toxicant-induced epigenetic alterations dose-dependent? Are these changes toxicant-specific? Are these modifications genome-wide or gene-specific? Finally, are the modifications accurate predictors of biological endpoints?

Among the frequently studied epigenetic signatures, microRNAs (miRNAs) have attracted a wealth of studies over the last decade that highlighted their importance, particularly in cancer development. This is likely due to the great versatility of these molecules as key regulators of gene expression in both physiological and pathological processes as one miRNA can target various mRNAs, and one mRNA molecule can be targeted by different miRNAs [22]. In cancers, many miRNAs have been identified to target mRNAs of oncogenes and tumor suppressors and consequently play major roles in cancer initiation, progression, and metastasis formation [23]. On the other hand, some oncogenes and tumor suppressors exert their actions by regulating specific miRNA expression. These findings have led researchers to determine the feedback mechanisms between miRNAs and their targets in different types of human cancers [24].

### 1.3. Epigenetics miRNA Signaling

With the rapid expansion of biological research and the development of advanced technologies, miRNAs have attracted more interest because numerous studies have demonstrated a significant association between miRNAs and various disease conditions. Generally, miRNAs are a category of endogenous, relatively small (~20–25 nucleotides), noncoding, single-stranded RNAs involved in gene expression at the post-transcriptional level by altering mRNA cleavage or translational expression [25]. Since the first discovery of miRNA about 20 years ago, researchers have discovered thousands of miRNAs in a wide variety of species. In plants, stressors will induce certain miRNAs which downregulate their corresponding mRNAs, leading to an increased level of beneficial mediators [26]. From a botanical perspective (plants, crops, agriculture, etc.), it is clear that regulation of miRNAs could lead to positive results including tolerance to drought [27], extremes in growth conditions [26], or resistance to metal toxicity [9,28,29]. Investigators are widely targeting miRNAs to promote the biosynthesis of therapeutically relevant compounds, such as vincristine and vinblastine production [30]. Concern rises from the fact that plant-food miRNAs are able to survive digestion and may subsequently affect gene expression in different organs of the consumer’s body [31].

Similarly, human miRNA functions by base-pairing with a complementary sequence in the mRNA; therefore, mRNA silencing can be achieved by either breakage of the mRNA, destabilization of the mRNA, or alteration of the translation process [32]. Due to the small size of miRNA, these molecules have been observed to fold back onto themselves, forming very small, tight, hairpin loop structures called pre-miRNA which are then exported into the cytoplasm where they undergo further processing into mature miRNA. Mature miRNA involved in gene silencing are incorporated into RNA-induced silencing complexes (RISC) [33]. At present, over 17,000 distinct mature miRNAs have been identified in over 140 species [34,35].

The location of miRNAs can be found throughout most human/mammal cells, and estimates have suggested that these miRNAs target approximately 60% of the mammalian genome [36,37]. Examination across a variety of species has resulted in observations that many miRNAs are highly conserved across species [25]. This conservation suggests that these miRNAs are involved in homeostatic biological functions [34] regulating the key events in the cellular homeostasis, including transcriptional and translational regulation of gene expression [34].

## 2. Incorporating Toxicoepigenomic Data into the Risk Assessment

In humans, it is now evident that environmental factors, including toxic metals, organic pollutants, and drugs, can exert influence on miRNA function [32]. Changes in miRNA expression levels are dependent on the toxicant to which the cells are exposed. For example, miR-146a is shown to be decreased following Cd exposure but increased following aluminum exposure [32]. Across the toxic metal spectrum, there are metal-specific variations in the miRNA response [32] which will be described in greater detail under the specific section for each metal.

Focused examination on environmentally relevant carcinogens has also demonstrated a wide range of miRNA-related changes that are dependent on the carcinogen in question [38]. The regulation of miRNAs by toxic metals is complex and intricate. The involvement of miRNA in tumor development appears to involve multiple miRNAs [38]. One miRNA, miR-17*, has been shown to inhibit the production and activity of mitochondrial antioxidant enzymes [39]. Reduction in antioxidant capabilities of the mitochondria leads to increased oxidative stress and inflammation, ultimately resulting in the development of cancer [40]. Recently there have been at least two studies that have followed cohorts for extended periods, attempting to correlate exposure to environmental pollutants (such as toxic metals) and the changes in miRNA expression [41,42].

Studies have found that the miRNAs play crucial roles at multiple stages of the biological processes, such as early cell growth, proliferation, differentiation, development, aging, and apoptosis. Additionally, more experiments have been implemented to show that miRNAs have connections with various human complex diseases. For example, miRNA-7a has clinical significance in human gastric cancer [43] Schulte et al. reported the capacity of miRNA-197 and miRNA-223 in predicting cardiovascular death and the burden of future cardiovascular events in a large cohort of coronary artery disease patients [44]. Furthermore, miRNAs may contribute to neurodegenerative diseases (NDDs) in response to environmental toxicant exposure via increasing oxidative stress and/or triggering inflammatory responses. Therefore, the miRNAs which are known to play a dynamic role in many biochemical pathways in the mammalian brain, including neuroplasticity, stress responses, cellular signaling, have become key players in the neurodegenerative phenotype of Alzheimer’s disease (AD), Parkinson’s disease (PD), and amyotrophic lateral sclerosis (ALS) [45].

**Figure 1:**
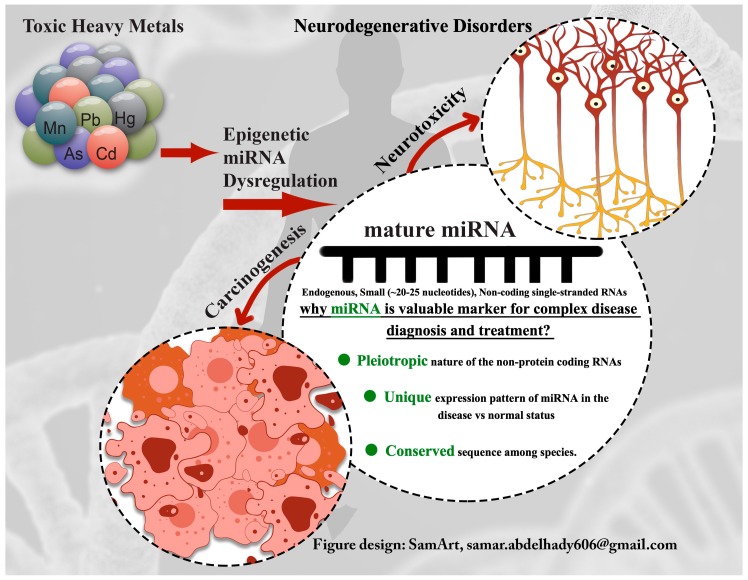
Toxic-metal-induced alteration in miRNA expression profile as a proposed mechanism for disease development.

The following text reviews the recent findings describing the mechanism of action of some toxic metals and discusses the risk of environmental exposure as a potential factor in accounting for the variability of disease progression. The graphical presentation of some of the toxic-metal-induced alterations in miRNA expression profile as a proposed mechanism for development of some diseases is given in Figure 1.

## 3. Determining Causal Toxic Metals and miRNA Signaling Cascade Associated with Disease Pathway

### 3.1. Cadmium-Associated Changes in miRNA Expression

In a recent study using a plant model, Jian et al., demonstrated that exposure to Cd for up to 3 days resulted in 39 differentially expressed miRNAs that were involved in homeostasis, stress response, transcription factor regulation, and secondary cellular metabolic responses [46]. Consequently, the ingestion of contaminated plants will result in higher concentration and bioaccumulation of Cd that contributes to increased Cd body burden. Hence, it is not surprising that significant efforts have been put forth to identify miRNA regulations that may lead to a reduction in Cd accumulation as well as reduction in the toxicity associated with Cd uptake [7,8].

One miRNA that was involved with reduced plant growth after exposure to Cd was miR-268 [47]. Elevation in the level of miR-268 was also associated with increased hydrogen peroxide and malondialdehyde (MDA) content, both indicators of elevated oxidative stress [47]. Additional miRNAs are being identified with each new study. In rice, a study aimed at finding novel miRNA after Cd exposure identified 19 new miRNAs associated with 11 unknown proteins [48]. For example, following exposure to Cd, there was an upregulation of miR-319 and miR-393 while miR-398 showed downregulation [49]. Other miRNAs involved in plant homeostasis are dependent on the location within the plant. In the roots, Cd exposure results in the downregulation of miR-390 [49]. Collectively, miRNAs are involved in processes that promote growth, minimize oxidative stress, and protect the plant.

As technology continues to improve, we will be able to identify additional miRNAs in plants that may be involved in regulatory responses following exposure to heavy metals. A similar response has been observed in aquatic life rather than plants. Exposure to Cd resulted in an inversely correlated relationship between miR-122 and metallothionein levels in farm-raised tilapia [50]. Cd exposure resulted in reduced expression of miR-122, leading to an increased metallothionein expression. Elevated metallothionein would be beneficial in attenuating the cellular stress caused by Cd exposure [50,51]. A similar study in *Daphnia pulex* demonstrated a positive correlation between miR-210 and hypoxia following Cd exposure [52]. Chen et al. propose that both Cd exposure and hypoxia will increase the generation of reactive oxygen species, triggering the increased activity of ERK, Akt, and hypoxia-inducible factor 1α (HIF1α), followed by promoting miR-210 expression [52].

The miRNA response to toxic metal exposure is no less complex in humans. Complicating data interpretation is the ability of Cd to exist in the body for years, in the range of 7–16 years [53] or even in some reports up to 45 years [54]. Even small exposures to Cd will result in a bioaccumulation in human tissues. As the tissues concentrate Cd, the toxic responses are elevated. Ovaries are one organ known to be susceptible to Cd accumulation and have a greater potential risk for toxicity either before or during pregnancy [55]. In ovarian granulosa cells, a 4-h exposure to Cd resulted in altered expression of five miRNAs involved in the kit ligand (kitl ) pre-mRNA alternative splicing [56]. Cd has been identified as a possible causative agent for preeclampsia, in which the ability of Cd to bioaccumulate increases its potential toxicity. Two miRNAs, miR-26a and miR-155, appear to be affected and may offer potential therapeutic targets in the future for preeclampsia treatment [57].

There is a strong link between Cd exposure and renal toxicity [14]. Investigators have tried to isolate changes in miRNA that could be used as potential biomarkers for cortical kidney injury [58]. Multiple miRNAs were altered following Cd exposure, which reduces the utility of changes in a single miRNA as a sole biomarker; however, a miRNA profile may be useful. Two miRNAs that displayed the largest (over 10-fold) increase compared to control were miR-34a-5p and miR-224-5p, whereas only miR-455-3p was reduced (52% decrease) [58]. Pellegrini et al. have reported temporal changes in miRNA expression that may be useful as biomarkers in identifying different phases of renal damage [59]. Again, this was following Cd exposure, but the two groups had very different miRNA alteration patterns. Pellegrini et al. have identified miR-18a, miR-132, and miR-146b as key miRNAs involved in responses following Cd exposure.

The abundance of miRNAs present can confound interpretation, yet the number of genes that are involved in cancer (392) and inflammatory responses (184) require a large number of miRNAs [60,61]. There are eight critical miRNAs involved with four cellular responses: inflammatory, cancer formation, cell death, and cellular growth and proliferation [60]. MiRNAs involved include miR-154, -222, -379, -204, -133, -10, and -375. Our understanding of the importance of miRNAs in cancer development has increased over the last few decades. The International Agency for Cancer Research (IARC) has classified Cd as a human carcinogen with a well-established association to kidney and prostatic cancers [62] as well as considerable involvement in other cancers, such as pancreatic cancer [63,64,65] and thyroid cancer [66], among which the role of miRNA in Cd-induced carcinogenesis is of great value. Evidence supporting the toxic metal modification of miRNA expression and cancer development is increasing, with Cd and As being two primary mediators [60,67]. Exposure to Cd leads to the transformation of prostate epithelial cells and a significant upregulation of KRAS (>20-fold) as well as the downregulation of 12 miRNAs, with miR-373 exhibiting the largest (11-fold) downregulation, and only a slight (6-fold) increase in miR-9 [68]. Cd exposure also can upregulate miR-372, which affects p21Cip1/WAF-1 expression in hepatic cell lines [69]. Changes in p21 activity will in turn alter p53 activity. The normal function of p53 in regulating apoptosis and cellular function and repair is necessary to maintain normal cell function and prevent cancer development. In bronchial epithelial cells, Cd exposure via cigarette smoking can modify multiple miRNA by both up- and downregulation [70].

Conclusively, these data demonstrate that Cd exposure is detrimental to normal cellular function and that exposure to Cd will result in both up- and downregulation of multiple miRNAs involved with normal cellular function, leading to the transformation of the normal cell and the subsequent development of cancerous cells. As a result of the aforementioned studies, as well as many others, it would be wise to incorporate epigenetic data into risk assessments. Incorporating epigenetic data would be particularly important for toxic metal exposures and would improve our ability to predict outcomes following exposure [19,71]. Some of the most important studies investigating the role of Cd on miRNA in in vitro and in vivo models are summarized in Table 1.

### 3.2. Arsenic-Associated Changes in miRNA Expression

Exposure to inorganic arsenic (iAs) is a global problem, as millions of people consume drinking water contaminated with naturally occurring iAs [72]. In these regions, As in drinking water usually exceeds the provisional guideline value of 10 µg/L [72]. Another source of growing concern for iAs exposure is through contaminated crops, particularly rice [73]. Rice (*Oryza sativa*) is generally grown in flooded soils under reducing conditions in which mobility of arsenite (As(III)) is higher than arsenate (As(V)), enhancing bioavailability of iAs to rice and leading to excessive iAs accumulation in rice grains and other plant parts (root and straw) [74]. Accumulation of iAs severely inhibits seed germination and plant growth [75].

Few studies have investigated the potential role of miRNA in plants in response to iAs exposure, but numerous differentially expressed genes have been identified. In roots of indica rice (*Oryza sativa* L. ssp. *indica*) under arsenite stress, 67 As-responsive miRNAs were recognized, belonging either to rice-specific miRNA families (e.g., miR-1861, miR-2121, miR-810, and miR-827) or to conserved miRNA families (e.g., miR-167, miR-169, and miR-390) [76]. In rice cultivar Nipponbare (*Oryza sativa* L. ssp. *japonica*) treated with arsenite, 36 new arsenite-responsive miRNAs were discovered, of which 14 seemed to be involved in gene regulation for transportation, signaling, and metabolism [73]. A total of 69 miRNAs belonging to 18 plant miRNA families had significantly altered expression in *Brassica juncea* exposed to arsenate, including miR-169, miR-172, and miR-390, also identified in the abovementioned arsenite-exposure studies [77]. Interactions between the hormones jasmonate and auxin were suggested to be involved in growth and signaling regulation under As stress. In arsenate treated maize (*Zea mays* L.) leaf, 22 (16 conserved and 6 novel) up- and 35 (33 conserved and 2 novel) downregulated miRNAs were identified [75]. A significant down- or upregulation in expression of miRNAs that regulate several developmental and metabolic processes, including signal transduction and photosynthesis, in response to arsenite or arsenate exposure was observed in these studies. The role of miRNA families in the regulation of hormonal biosynthesis or function of jasmonates (miR-319) and auxin (miR-160 and miR-167) during plant exposure to arsenite [73] or arsenate [77] has also been indicated.

In addition to regulating growth and development, the most important function of miRNAs in plants is the regulation of cellular processes related to plant adaptation to As-induced stress. Moreover, the obtained results indicate that rice invests more energy and resources into immediate defense needs than into normal growth requirements [73].

Based on sufficient evidence for carcinogenicity, inorganic As compounds are classified by IARC [78] as “human carcinogens”. Several epidemiological studies confirmed the relationship between chronic exposure to elevated levels of As from drinking water and skin, lung, bladder, liver, kidney, and pancreatic cancers [79,80]. iAs is genotoxic and affects cellular signaling, cellular proliferation, DNA structure, epigenetic regulation, and apoptosis [81,82]. Previous work has demonstrated that As can alter miRNA expression patterns in in vitro and in vivo models of As-induced carcinogenesis. Both up- and downregulated miRNAs have been associated with cancer, acting either as oncogenes, tumor suppressors, or both [83,84,85]. miR-182-5p suppression was shown to contribute to hypoxia-inducible factor HIF2α overexpression in response to arsenite exposure, suggesting that aberrant overexpression of HIF2α via miRNA dysregulation is involved in As-induced carcinogenesis [86]. After As exposure, analysis of miR-200 family members, specifically miR-205, indicated that deregulated miRNAs could be potential biomarkers for As exposure and could be used as diagnostic markers for the formation of early urothelial carcinoma [87]. Several studies have focused on miRNAs as promoters of the apoptosis induced by arsenic trioxide, which is commonly used in the treatment of acute promyelocytic leukemia, mainly supporting the hypothesis that miRNAs may play a mediatory role in eliciting the multitarget action of this compound [88,89].

Adverse health effects of As have not been limited to its carcinogenicity [90]. Long-term exposure to iAs, mainly via drinking water, affects almost every organ system in the body, including the brain. As ranked first among toxicants posing a potential threat to human health based on known or suspected toxicity that contributes significantly to human chronic diseases such as diabetes mellitus (DM), hypertension (HTN), cardiovascular diseases (CVD), and neurological disorders [91]. Recent studies have revealed that low concentrations of arsenic impair neurological function, particularly in children. Epidemiological literature report arsenic neurotoxicity in children and adults with emphasis on the cognitive dysfunction, including learning and memory deficits and mood disorders. It has been challenging to elucidate the mechanisms of As toxicity following long-term exposure due to confounding factors of co-exposure to other environmental pollutants and lifestyles associated with low socioeconomic status (SES) [92]. Hence, As accumulation and subsequent toxicity is likely mediated through multiple mechanisms of action. These include the depletion of methyl groups affecting epigenetic profiles, the uncoupling of oxidative phosphorylation and increased ROS, the inhibition of thiol-containing enzymes and proteins, altered signal transduction and cell proliferation, and reduced DNA repair inducing genotoxicity. Additional mechanisms of As-mediated toxicity in the brain have gained significant interest recently, including hippocampal dysfunction; glutamatergic, glucocorticoid, cholinergic, and monoaminergic signaling pathways; synaptic plasticity, and neuronal epigenetic pattern alteration after arsenic exposure [92].

There have been insufficient human population-based studies examining the relationship between As exposure and altered miRNA expression and the potential for these modifications to result in disease development. Ruiz-Vera et al. [93] have demonstrated an alteration in the expression levels of two miRNAs (miR-155 and miR-126) associated with cardiovascular disease in women in Mexico exposed to iAs via drinking water. Similarly, high iAs exposure was associated with altered profiles of circulating miRNAs in plasma of healthy subjects from Mexico, and four of the identified miRNAs (miR-423-5p, -423-5p +1, -142-5p -2, and -454-5p) appear to be linked to a risk of cardiometabolic diseases [94]. In a study that investigated the health impacts from prenatal exposure ranging up to 236.0 μg As/L in drinking water in Mexico, researchers found a set of differently expressed miRNAs and mRNAs that were implicated in innate and adaptive immune response [95]. Specifically, As-associated changes in miR-20b and miR-107 were observed, both of which have been associated with diabetes mellitus [96].

The levels of miR-21 were upregulated in individuals from the highly As-contaminated district of West Bengal where, within the exposed group, miR-21 expression levels were higher in the individuals with skin lesions when compared with the individuals without skin lesions [97]. The results of a meta-analysis conducted by Liu et al. [98] showed that exposure to As increases miR-21 levels and reduces the levels of tumor suppressor genes PDCD4, PTEN, and Spry1, ultimately leading to the malignant proliferation of cancer cells. It has been shown that As-mediated skin cancer has different miRNA expression profiles than sunlight-induced skin cancer, suggesting that these miRNAs are potential biomarkers and therapeutic targets for As-induced skin tumors [99].

Mammalian studies have suggested a role for miRNAs in NDDs and neuroprotection. Workers in Paul Greengard’s laboratory used transgenic mice to support the hypothesis that miRNA function is essential for mammalian neuronal survival and that, conversely, compromising miRNAs causes neurodegeneration [100]. In this study, the cerebellar Purkinje-cell-specific Pcp2 promoter was used to drive a Cre recombinase causing the dicer gene (and the production of most mature miRNAs) to be knocked down beginning in the second week of life with Pcp2 gene activation. Loss of cerebellar expression of both dicer and most mature miRNAs is followed by the neuropathological death of Purkinje and dendritic cells, together with an ataxia phenotype in the mice. The authors hypothesize that, as dicer downregulation induces neuronal cell death, then some human neurodegenerative diseases may be caused by loss of small regulatory RNA molecules. As dicer is knocked out in the cerebellum, the authors conclude that their work may be relevant to spinocerebellar ataxias.

Two other studies from Walter Lukiw’s laboratory analyzed DNA arrays to evaluate the expression of a subset of 12 miRNAs in the Alzheimer’s disease (AD) hippocampus in comparison with nondemented controls and fetal brain. The expression profiling results demonstrated elevated miR-9 in both fetal and AD hippocampus, elevated miR-128a in AD only, and only slightly increased miR-125b in AD brains [101]. The same research group performed a second study using cultured human fetal-brain-derived primary neural (HN) cells containing both astrocytes and neurons which were treated with metal salts, such as aluminum and iron sulfates known to stimulate the generation of ROS. The RNA from HN cells was compared to AD brains using the same DNA miRNA array from the previous study. Remarkably, the HN cells responded similarly, with nearly identical changes (increased expression of miR-9, miR-128 and, to a lesser extent, miR-125b) vs. controls, as were seen in the AD brain in comparison with nondemented brains. These data provide evidence in accordance with the hypothesis that ROS influence AD brain through pathways specifically mediated by miRNAs. Furthermore, miR-125b, which trends higher in both AD brain and in ROS-treated HN cells, is predicted to target the mRNA of synapsin I and synapsin II, and thus downregulation of synapsins in AD may be partially explained by miR-125b upregulation [101,102].

These results strongly support the critical role of the dysregulated miRNA expression in As-induced carcinogenesis as well as in several other chronic diseases. Recently published studies pertaining to the roles of miRNAs in NDDs—including Alzheimer’s disease and Parkinson’s disease—describe the involvement of miRNA, although few have connected these roles to the influence of toxic metals on miRNA regulation. Given the fact that As exposure continues to represent a major public health threat for populations worldwide, further studies are necessary to evaluate the vital modification during As-associated transformation. Future studies should highlight the mechanisms by which As can perturb miRNA expression and determine which biological effects are related to the altered miRNA expression. Some of the most important studies investigating the role of this metal on miRNA in in vitro and in vivo models are summarized in Table 2.

### 3.3. Lead-Associated Changes in miRNA Expression

There is growing recognition of the impact of environmental toxic metals, particularly Pb, on the epigenetic regulation of gene expression [103]. To date, few studies have connected Pb toxicity to changes in the short- and long-term expression of miRNAs that target epigenetic mediators and neurotoxicity.

Pb toxicity can be observed in all living organisms, including plants, animals, and humans. *Platanuss acerifolia* is a widely cultivated avenue tree generally resistant to pollution in urban regions. The influence of Pb on *P. acerifolia* miRNA expression has been examined by Wang et al., with 55 known and 129 new miRNAs identified as involved in the Pb-induced stress response [104]. Another experiment performed on *Raphanus sativus* identified 25 known and 9 novel Pb-responsive miRNAs primarily involved in the stress-related signal, metal absorption, and homeostasis mechanisms. The authors observed the downregulation of PN-miRNAc1, which plays a role in ROS signaling and synthesis of the glutathione S-transferase [105]. Additionally, numerous miRNAs were identified in plants after exposure to Pb. He et al. found the downregulation of miRNA398 expression and upregulation of miRNA159, miR162, and miR396 expression in cotton leaves after Pb exposure. On the other hand, ten miRNAs (miR159, miR162, miR169, miR395, miR396, miR397, miR398, miR833a, miR858a, and miR5658) were upregulated and two miRNAs (miR156 and miR396) were downregulated in cotton roots [106]. These findings show that there is a complex response in plants upon Pb exposure, and further studies should be performed to better understand the molecular mechanisms of Pb-induced stress in plants.

In animals and humans, the toxic effects of Pb have been extensively documented. Masoud et al. observed a decrease in miRNA-106b and miRNA-124 at postnatal day 700 in the hippocampus of mice treated with Pb acetate for the first 20 days after birth. They also showed that miRNA-29b and miRNA-132 concentrations increased by at postnatal day 20, while the concentration of miRNA-34c decreased at postnatal day 180. Selected miRNAs target the mRNAs for transcription factor protein 1, amyloid-β precursor, microtubule-associated protein tau, and other proteins that may play a role in the pathogenesis of AD. These finding indicate that early life exposure to Pb alters the expression of specific miRNAs that target synthesis of neurotoxic proteins in later life [107]. In the 8 week study performed with Sprague-Dawley rats exposed to Pb, the authors reported an increase in the expression of miR-204, miR-211, miR-448, miR-449a, miR-34b, and miR-34c and a decrease in expression of miR-494 in the hippocampus [108]. An in vitro study performed on immortalized murine choroidal epithelial cells Z310 as a model for blood–cerebrospinal fluid barrier showed that Pb increased miR-203 expression and regulated tight junction targeting of the tricellulin protein, leading to increased cerebrospinal fluid barrier permeability [109].

Lead may pass through the placenta and alter fetal development. The human nervous system is sensitive to the toxic effects of many external toxicants during the course of all three trimesters, as well as many years after birth. Li et al. showed that Pb levels had a positive association with miR-146a, miR-10a, miR-190b and miR-431 expression and a negative association with miR-651 expression in the placenta [41].

The possible usage of miRNAs as a sensitive biomarker of toxicity or disease prediction has gained much importance in recent years [110]. In a case-control study in China, including 2115 Hong Kong adolescents, change in miRNA-21 content was associated with measured urinary Pb levels and albuminuria. The correlation between miRNA-21 and urinary Pb suggests that miRNA-21 might be involved in the pathogenetic mechanism of albuminuria. Lead levels also correlate with miRNA-221, but there was no difference in miRNA-221 urine levels between control and albuminuric groups [42]. Moreover, research has shown that miRNA-21 overexpression has been found in many cancer tissues, implying that miRNA-21 has a regulatory function in carcinogenesis and may act as a proto-oncogene [111].

Lead continues to be a major occupational toxicant, and widespread usage in industry has led to an increased level of environmental Pb contamination and exposure. The differences in expression of six miRNAs were identified in plasma between workers with high and minimal Pb exposure. Namely, downregulation of hsa-miR-572 and hsa-miR-130b and upregulation of miR-520c-3p, miR-148a, miR-141, and miR-211 were observed in higher exposed participants. From all of these miRNAs, the authors identified miRNA-211 as a potential biomarker in plasma for Pb-exposed workers [103].

Additionally, occupational inhalation exposure to Pb is positively correlated with miR-222 expression and negatively correlated with miR-146a in peripheral blood leukocytes, which are related to oxidative stress and inflammatory processes [112]. In the study performed on 145 male subjects exposed to several toxic metals, including Pb, miRNA-21-5p, and miRNA-122-5p were found to be higher than in the control nonexposed group [113]. Undoubtedly, Pb exposure is associated with alteration in miRNA expression in a complex manner that necessitates further studies to elaborate on the exact molecular mechanism and consequences of expression of miRNA following Pb exposure.

### 3.4. Mercury-Associated Changes in miRNA Expression

Mercury is one of the most extensively used metals in industry and is used primarily in mercury thermometer and barometer production and gold extraction. These toxic metal can still be found in many scientific instruments and has been used extensively in switching mechanisms. One aspect of Hg toxicity studies has been the differentiation between organic and inorganic forms. The organic form (methyl Hg, etc.) is readily absorbed through the skin and gastrointestinal tract. Inorganic Hg (HgCl_2_, etc.) has a lower toxic potential and is absorbed less through skin but can still be absorbed via the gastrointestinal tract following ingestion [114].

Many mechanisms have been proposed for Hg toxicity [12,13,115,116,117,118,119,120]. Of interest is the ability of Hg to increase free radical generation and bind to thiol groups on proteins [121,122,123,124]. Since different chemical forms of Hg are found ubiquitously throughout the environment, the ability of Hg to enter plants via the root system and eventually induce toxicity is an area of major research interest [47]. Hg-contaminated soil has been shown to reduce the growth and yield of many crops [9]. Along with crop reduction, Hg exposure alters the expression of miRNA in both an up- and downregulatory fashion [9]. In some instances, most of the miRNA work was done simply to map the miRNA family and the corresponding genome of a particular plant species. Hg exposure in *Medicago truncatula* resulted in a specific induction of at least 12 miRNAs [125]. Of the upregulated miRNAs, 11 were unique to the Hg-treated plants, suggesting that there are Hg-specific miRNAs that respond to Hg exposure [125]. Researchers proposed that if an altered profile can be identified, this profile might be used as a biomarker for exposure to heavy metals. The toxicity of Hg in humans has been well studied, from the description of “mad hatter disease”, where Hg-containing solutions utilized to treat the material used in hat-making resulted in mental changes in the hat maker, to current Hg-mediated cellular changes. Human exposure to Hg has been reported to be involved in the development of many cancers [1,2,40].

One study examined the circulating miRNAs found in plasma in workers who were exposed to Hg in the workplace. RT-PCR analysis revealed that two miRNAs (miR92a-3p and miR486-5p) in plasma were significantly upregulated in the Hg-poisoned group compared to controls [126]. The role of the miRNAs following Hg exposure is unknown. The authors report that changes in miR92a-3p and miR486-5p may not be due to Hg exposure but may be due to a secondary mechanism and that more work is necessary to accurately correlate Hg exposure to miRNA changes in humans [126].

Examining in utero exposure to Hg by measuring changes in miRNA expression in the placenta did not find the same strong correlations for Hg exposure as with other heavy metals. Instead, a downregulation of multiple let-7 family members was reported, which correlated with elevated concentrations of Hg [41,71]. From earlier studies, the proposed mechanisms for mercury toxicity involve binding to sulfhydryl groups on proteins, increasing oxidative stress, and reducing oxidative defense. However, these mechanisms cannot fully explain the damage to numerous organic systems that has been associated with mercury exposure. Finding a reliable profile of miRNA changes would significantly aid in risk assessment for toxic metal exposure [19].

### 3.5. Manganese-Associated Changes in miRNA Expression

Mn is one of the most abundant, naturally-occurring metals in the environment. Mn in trace amounts is also essential for normal physiological function and is used as a cofactor for enzymes such as manganese superoxide dismutase (MnSOD). In elevated concentrations, Mn is a highly toxic transitional metal. The dopaminergic system is very sensitive to Mn toxicity and is a target for elevated Mn concentrations in welders using Mn-containing flux [127]. Mn toxicity involves the generation of free radicals and alterations in normal mitochondrial function [2,128,129].

Similar to other metals, in high soil concentrations Mn will accumulate in various parts of the plant and elicit a specific pattern of toxicity that is dependent on its location. miRNA expression analysis in *Phaseolus vulgaris* demonstrated a pattern of multiple miRNA expression changes [130]. In general, a similar pattern of change was observed following Mn exposure as was observed following exposure to other metals, suggesting similar stressor-induced pathway activation. Stress response miRNAs such as miR319 and miR398 were upregulated under most stressor conditions. A large portion of the work examining Mn-induced toxicity has focused on the central nervous system due to the sensitivity of the dopaminergic system to Mn-induced toxicity [127]. In vitro studies have revealed a complex Mn response in neuronal cultures. miRNAs regulating proinflammatory responses, such as TNFα and IL-6, show a downregulation promoting inflammation [131]. Since Mn exposure may result in a Parkinson’s-like syndrome referred to as “manganism”, in vitro work has examined the effects of Mn on cellular toxicity and regulation of the expression of proteins known to be altered in Parkinson’s disease such as α-synuclein. He et al. showed that the altered expression of hsa-miR-4306, which targets PARK9, a suppressor of α-synuclein production and activity [132,133]. Further work investigating the ability of Mn to cause overexpression of α-synuclein, leading to neuronal damage, showed a downregulation of miR7 and miR433, suggesting that these two miRNAs were important for the protection of neuronal cells after Mn exposure [134,135]. However, another study reported that 12 of 43 miRNAs examined after Mn exposure were significantly elevated compared to controls [135]. Collectively, Mn exposure will alter miRNA expression and release in a manner dependent on the miRNA. Some miRNAs will demonstrate elevated expression; others will be significantly downregulated. The response will be dependent on what gene and response the miRNA is regulating.

Outside the central nervous system, Mn exposure is understood to be a carcinogen and has been implicated in multiple forms of cancer [136,137,138,139]. In prostate cancer cells, it was found that an upregulation of miR17* following Mn exposure yielded a protective effect in PC-3 cells, significantly reducing the tumorigenicity of the cell line [39]. From the current literature, it is clear that elevated concentrations of Mn will elicit cellular toxicity. Previously, we understood Mn toxicity to involve the generation of free radicals, alteration of normal mitochondrial function, and generation of proinflammatory mediators. Currently, more emphasis has been directed toward the ability of Mn exposure to exert a complex pattern of miRNA expression changes. Further work is needed to improve and increase our understanding of the intricacies associated with Mn-mediated miRNA changes.

Some of the most important studies investigating the role of Pb, Hg, and Mn on miRNA in in vitro and in vivo models are summarized in Table 3.

## 4. Therapeutic Implications of miRNA in Human Diseases

miRNAs hold great promise from a therapeutic point of view, especially with the proven ability of a single miRNA to influence several target genes, making it possible to potentially modify a whole disease phenotype by modulating a single miRNA molecule. Hence, alongside the exponential increase in the number of studies crosslinking toxic-metal-induced miRNA alterations to disease development, great strides have been made in targeting miRNA expression for prevention and potential treatment of disease. One strategy is the use of oligonucleotides or virus-based constructs to either directly block the expression of a disease-associated signature miRNA or to directly substitute for the loss of expression of the miRNA. The other strategy is based on indirectly employing drugs to alter miRNA expression by targeting their transcription and processing. Blocking miRNA expression can be achieved by the use of antisense oligonucleotides, miRNA sponges, miRNA masks, and small RNA inhibitors. Restoring downregulated miRNA expression can be achieved by using the so-called miRNA mimics, which are synthetic miRNA molecules, or by inserting genes coding for miRNA into viral constructs. The small-molecule miRNA inhibitors can be employed at the transcriptional level to prevent the transitions from DNA transcript to pri-miRNA and pre-miRNA. At the mature miRNA level, antisense oligonucleotides can be employed to induce degradation of the mature miRNA into a duplex form with the antisense oligonucleotide. At the functional level, miRNA masks can bind complementarily to the 3′ UTR region of target mRNA, competing for bindings with endogenous miRNAs for the specific target. miRNA sponges can be employed to bind target miRNA via complementary mRNA binding sites, decreasing expression levels of target miRNAs [140].

The functional effect of miRNAs differs depending on their expression levels. They have either an oncogenic potential or tumor-suppressor effect depending on their downstream impact on target genes and thereby control the biologic manifestations of cancers. The activity of a lost or downregulated tumor suppressor miRNA can be restored by using miRNA mimics [141]. In this context, there are some miRNA-based trials for treatment of cancers; for example, miR-34 is one of the tumor suppressor miRNAs, and it is significantly downregulated in many kinds of cancer. Consequently, a cancer therapy synthetic miR-34 (MRX34) entered phase I clinical trial for liver cancer and metastasis from other cancers (NCT01829971) [142]. Similarly, miR-27a has been described to be a likely targeted therapy for lung cancer [143]. miRNA-loaded minicells (miR-16-based mimic miRNA) are designed to counteract the loss of the miR-15 and miR-16 families and are used in clinical trials for small-cell lung cancer and mesothelioma [144]. The miR-205BP/S3 is a promising possible therapeutic modality for melanoma [145]. Let-7 is well recognized as one of the important tumor suppressors. Re-expression of the tumor-suppressor let-7 is another proposed miRNA therapeutic strategy to upregulate tumor-suppressor miRNA by exogenously transfecting with pre-let-7, which leads to the inhibition of growth [140]. In addition to tumor suppressor miRNAs, some of the miRNAs can serve as oncogenes and can be used as therapeutic targets for cancer, including miR-21, which is significantly overexpressed in many types of human cancers; thus, miR-21 is a potential therapeutic target for a certain cancer [146].

## 5. Conclusions and Future Perspectives

Environmental and occupational exposure to toxic heavy metals can alter epigenetic regulatory signatures such as DNA methylation, histone modification, and microRNA (miRNA) expression patterns. miRNAs are endogenous, noncoding RNAs of approximately 25 nucleotides, which play fundamental gene-regulatory roles by binding to the 3′ UTR of protein-coding genes to mediate their post-transcriptional repression. As circulating miRNAs are in a remarkably stable form that is protected from endogenous RNase, serum or plasma miRNAs have been widely used as blood-based markers for diagnosis of human disease. These early studies attempting to identify miRNA biomarkers have focused only on a select few diseases and have not expanded to include responses following exposure to environmental toxicants. At present, the expression patterns of miRNA and their susceptibility to environmental toxicant factors are a focus of environmental epigenetics. Unfortunately, insufficient studies illuminating the underlying mechanisms have been published to further our understanding. Establishing a reliable profile of miRNA changes would significantly support the risk assessment of toxic metal exposure and would be tantamount to developing altered miRNA profiles as potential biomarkers following metal exposure. A well-defined miRNA profile might be used as a biomarker for diagnosis of human complex diseases such as cancers, NDDs, and many other chronic diseases that have been associated with environmental toxic metal exposure. In essence, the profile of miRNA changes associated with toxicant exposure would act as a molecular fingerprint to help identify the responsible toxicants.

Identifying disease-related miRNAs is essential to the treatment, diagnosis, and prevention of a variety of clinically relevant diseases. However, identifying the associations between miRNAs and diseases with routine experimental methods is expensive and time-consuming. With the development of biological technology, many experiments have been implemented to produce vast numbers of miRNA-associated datasets. Based on the assumption that functionally similar miRNAs are more likely to have associations with phenotypically similar diseases, many computational approaches have been introduced for the identification of miRNA–disease associations. Therefore, it is essential to develop new computational tools for potential miRNAs–disease association prediction. We additionally emphasize the need for more descriptive and “lifelike” experimental disease models of human complex disorders, such as NDDs, in order to identify candidate biomarkers and pinpoint susceptible groups or life stages to be translated to expansive prospective studies within the exposome framework.

## Figures and Tables

**Table 1:** Summary of studies analyzing effects of Cd on microRNAs (miRNAs).

Type of Study	Cell Culture/Species	Treatment Doses and Duration	Effects on miRNAs	Ref.
In vitro	Murine ovarian granulosa cells	10, 20 and 40 µM 2, 4, 6, and 8 h	Target gene function of 29 miRNAs mainly consisted of cell metabolism regulation, mRNA post-transcriptional regulation, IL-6-mediated signal transduction, cell cycle, proliferation, differentiation, migration. These miRNAs are associated with target genes associated with the signaling of Ras, Rap1, Foxo, Hippo, MAPK and carcinogenic pathway, actin cytoskeleton regulation, stem cell signaling pathway polymorphism and local adhesion resulting in cell division and tumorigenesis.	[56]
In vitro	Primary human proximal tubular epithelial cells (HPTECs)	25 µM CdCl_2_ 6 and 24 h	Increased expression of miR-132-3p after 6 h. Increased expression of miR-132-3p after 24 h. 10-fold increase in miR-34a-5p and miR-224-5p expression; decreased miR-455-3p expression. Decreased expression of miR-18a-5p and miR-146b-5p after 24 h. Temporally increased miR-132-3 and changes in other miRNA expression may be useful in assessing renal damage.	[58,59]
In vitro	Cd-transformed prostate epithelial cells (CTPE) developed from immortalized nontumorigenic human prostate epithelial cells (RWPE-1)	10 µM 8 weeks	12 miRNAs were downregulated; three miRNAs were upregulated. Increased oncogene mRNA expression (KRAS by 2000%; RAB22A by 48%). Increased cell signaling E2F1 mRNA by 52%. Decreased cell-adhesion-related genes CADM1 mRNA by 65% and CTNNA1 mRNA by 25%. Increased cell survival and apoptosis-related genes BCL2L1 mRNA by 25%; decreased p27^Kip1^ protein by 49% and FOXO4 mRNA by 61%. Increased expression of RAS/ERK pathway activation. Following exposure to Cd, transformation of epithelial cells resulted in increased tumorigenic cell formation.	[68]
In vitro	Hepatoma cell line (HepG2)	0.1–10 µM CdCl_2_ 24, 48, and 72 h	No cell cycle arrest was observed. The distribution of cell population among the cell cycle phases (G1, S, G2/M) did not change. KEGG mapping demonstrated that the p53 gene was not regulated at the transcriptional level, nor was there clear evidence of an upregulation of this transcription factor. The p53 was translocated into the nucleus, yet p21^Cip1/WAF-1^ was not activated. Mir-372 able to affect p21^Cip1/WAF-1^ was upregulated. Changes in p21 activity will alter p53 activity, and p53 is critical for maintenance of normal cell function.	[69]
In vivo	Male Sprague-Dawley rats	s.c. 0.6 mg/kg CdCl_2_ 12 weeks	Expression levels of 44 miRNAs were significantly increased (miR-21-5p, miR-34a-5p, miR-146b-5p, miR-149-3p, miR-224-5p, miR-451-5p, miR-1949, miR-3084a-3p, miR-3084c-3p). Expression levels of 54 miRNAs were significantly decreased (miR-193b-3p, miR-455-3p, miR-342-3p).	[58]

**Table 2:** Summary of studies analyzing effects of As on miRNAs.

Type of Study	Cell Culture/Species	Treatment Doses and Duration	Effects on miRNAs	Ref.
In vitro	Human renal epithelial cells (HK-2)	2.0–5.0 µM NaAsO_2_ 30 weeks	mRNA of HIF1α decreased to 44.7% of control at 30 weeks. mRNA of HIF2α increased to 144% of control at 30 weeks. mRNA of CPT1A decreased to 43.2% of control at 30 weeks. HIF1α protein expression not changed. HIF2α protein expression at the protein level was overexpressed. CPT1A was decreased at protein level. Inactivation of Von Hippel–Lindau and impaired protein degradation of HIF2α were not observed. Levels of miR-142-5p increased (327.3%). Levels of miR-182-5p and miR-802 were 42.4% and 54.0% of control, respectively.	[87]
In vitro	Pancreatic cancer cells Panc-1 and Patu8988 cells	3 µmol/L As_2_O_3_	Increased expression level of miR-330-5p.	[89]
In vitro	Normal human urothelial cells (HUC1)	1 µM As_2_O_3_ 2, 4, 6, 8, and 10 months	Increased growth and cellular proliferation. Increased expression of p-AKT, m-TOR, and p-PI3K. Increased expression of p-EGFR, ERK, and cyclin D3. Reduced expression of miR-200a, miR-200b, and miR-200c. No change or slight overexpression was observed for miR-205. Decreased E-cadherin protein expression.	[88]
In vivo	Patients with urothelial carcinoma (urine samples)		Low levels of miR-200a, miR-200b, miR-200c, and miR-205. Decreased expression of miR-205.	
In vitro	HaCaT cells (immortalized human keratinocytes)	0.5 ppm 15 passages 60 days	Increased expression of miR21. Decreased expression of PTEN and PDC4. Increased expression of survival proteins pAKT and PI3K and increased cell survival.	[98]
In vivo	Peripheral blood mononuclear cells Chronically exposed humans with skin cancers (SCC and BCC)		Expression of miR21 was 2.9-fold upregulated vs. unexposed. Expression of miR21 was 4.49-fold upregulated vs. without skin lesion.	
In vivo	Keratinocytes Chronically exposed humans with skin cancers (SCC and BCC)		miR-425-5p and miR-433 were induced. miR-184 and miR-576-3p were induced in SCC relative to BCC. miR-29c, miR-381, miR-452, miR-487b, miR-494, and miR-590-5p were selectively suppressed in BCC relative to SCC.	[100]

**Table 3:** Summary of studies analyzing effects of Pb, Hg, and Mn on miRNAs.

Toxic Metal	Type of Study	Cell Culture/Species	Treatment Doses and Duration	Effects on miRNAs	Ref.
**Pb**	In vivo	Male pups from C57BL/6J strain mice	0.2% Pb acetate via mothers’ milk Postnatal days (PND) 1–20 days	Increased expression of miR-106b (1.5-fold) at 20 PND but decreased at 20–180 PND. Decreased expression of miR-124 at PND 700. Increased expression of miR-29b (1.6-fold) and miR-132 (4.8-fold) on PND 20. Expression of miR-34c decreased at PND 180. Expression of miR-148a remained unchanged.	[108]
	In vivo	Male Sprague-Dawley rats	100, 200, and 300 ppm Pb acetate 8 weeks	Upregulated miR-204, miR-211, miR-448, miR-449a, miR-34b, and miR-34c. Downregulated miR-494.	[109]
	In vivo	Battery factories workers (blood samples)		Upregulation of miR-520c-3p, miR-148a, miR-141, and miR-211. Downregulation of miR-572 and miR-130b.	[104]
**Hg**	In vivo	Mercury thermometer factory, female workers (blood samples)		Upregulation of miR-92a-3p, miR-122-5p, miR-451a, and miR-486-5p. Downregulation of miR-16-5p, miR-30c-3p, miR-181a-5p, and let-7e-5p.	[127]
**Mn**	In vitro	Human neuroblastoma cells SH-SY5Y	2 mM MnCl_2_ 24 h	44 miRNAs increased expression (miR-210-3p, miR-320b, miR-502-3p, miR-193a-3p, miR-192-5p, miR-4508, miR-4306, and miR-7704). 29 miRNAs decreased of expression (miR-151b and miR-877-5p). Decreased expression level of ATP13A2, which is involved in the transport of divalent cations. Downregulation may protect against Cd-related toxicity but will potentiate Mn-induced toxicity in human neuronal cells.	[134]
